# Oral Cancer and Twitter: An Analysis of Oral Cancer Awareness Month Tweets

**DOI:** 10.7759/cureus.54055

**Published:** 2024-02-12

**Authors:** Nada Binmadi

**Affiliations:** 1 Department of Oral Diagnostic Sciences, King Abdulaziz University, Jeddah, SAU

**Keywords:** awareness, analysis, social media, twitter, oral cancer

## Abstract

Objective: The objective of this research was to assess Twitter usage during Oral Cancer Awareness Month and explore the content and engagement related to oral cancer.

Methods: A comprehensive search was performed using relevant hashtags and keywords related to oral cancer on Twitter throughout the oral cancer awareness month, April 2022. All extracted tweets that match the inclusion criteria were analyzed for content, users were classified, and their countries were identified.

Result: A total of 5551 English tweets were identified during Oral Cancer Awareness Month, and 5543 were included in the analysis covering a wide range of oral cancer-related topics. The analyzed tweets encompassed a diverse range of topics, from cancer and oral health to oncology, cancer research, cancer awareness, and even discussions related to alcohol. We found that the majority of users who post on Twitter were individuals. The most common tweets were posted from the USA.

Conclusions: This study provides an analysis of Twitter activity during Oral Cancer Awareness Month, highlighting the diverse range of content being shared, offering valuable insights. The findings demonstrate the importance of leveraging social media platforms to disseminate information and raise awareness. With a strategic approach to social media, organizations and individuals worldwide have the power to amplify their message, attract attention, and effectively advocate for oral cancer awareness.

## Introduction

Malignancies that develop in the mouth, oropharynx, nasal cavity, and paranasal sinuses are referred to as head and neck cancers, which are the sixth most frequently diagnosed type of cancer [1]. The history and physical examination of the patient are very important in the prompt diagnosis of oral cancer. Biopsy specimens are used to identify or rule out malignancy and are usually performed in suspected patients. With this in mind, patients should be aware of the clinical features of oral cancers so that they can be identified and managed early. Tobacco use is thought to be a major risk factor responsible for head and neck cancers worldwide [2,3]. However, recent studies conducted on dental patients reveal that they have little understanding of the indicators and manifestations of oral cancer, indicating a need to foster greater public awareness and leading to a considerable recent surge in related health-care programs [1,3-8]. These initiatives are typically held in departmental stores and other places where people enjoy their leisure time. Because oral cancer can be avoided by minimizing risk factors and addressing them in their earliest phases, increasing public understanding and awareness would improve both primary and secondary prevention [8].

Oral cancers are a public health concern and need to be addressed promptly. April is Oral Cancer Awareness Month, and cancer coverage in the media has played an important role in raising awareness [9]. The goal of promoting oral cancer awareness is to enlighten the general population on the risk factors and oral manifestations, as well as the need for regular dental check-ups and early identification. Awareness is critical because oral cancer is often undiscovered until it has evolved to a more advanced stage, making treatment more difficult. Clinical detection and examination of oral mucosal lesions have the potential to discover and treat almost all oral premalignant conditions and cancers [10]. By raising knowledge of oral cancer, health-care practitioners, lawmakers, and the public can collectively encourage early identification and enhance the effectiveness of treatment, leading to overall improvement in the quality of life for people living with the disease [11].

Individuals now have access to a variety of digital networks for discovering and exchanging health information. As a result, traditional media is no longer the only way to communicate cancer awareness, as various stakeholders such as physicians, health-care organizations, and patients use social media to spread disease knowledge and awareness, especially the younger generation. Social media platforms such as Facebook, Twitter (now X), and Instagram can be powerful tools for raising awareness of oral cancer [12]. By sharing information and educational materials, individuals and organizations can educate the public about the signs and symptoms of oral cancer, underscore the importance of early detection, and highlight available treatments. The impact of these tools is not yet clear [13].

According to one recent study, the use of social media as a viable means of encouraging initiatives to enhance public health and dental education is important. These platforms are simple to use and popular among people of various socioeconomic backgrounds and have been shown to result in good short-term retention of oral health knowledge. This may provide better insight into future investments in such treatments [14]. Similarly, Twitter is a prominent social media application that individuals can use to express their views on certain subjects, opinions, responses, and so on in around 10,000 characters. Users can follow one another to real-time feeds of each other’s posts. Individuals may retweet other people’s tweets and make specific references to others by their user names. According to researchers, 8.85% of adults who go on the internet have Twitter accounts [15,16]. With its interactive platform, Twitter can be used to spread awareness, provide support, and share information about oral cancer and other diseases.

A number of studies demonstrate the significant contribution of Twitter data in the context of monitoring, identification, and forecasting public health issues. Twitter data has been useful in helping with public health activities such as disease monitoring, detection of incidents, pharmaceutical surveillance, predictions, disease monitoring, and geographical verification [17]. Previous studies have shown that material published on social media sites such as Twitter provides vital insights into how people know and think about health topics [18,19]. Different studies suggest that Twitter as a platform can raise awareness of oral health and oral cancers. This study aims to examine the content and engagement and interaction surrounding oral cancer during the designated Oral Cancer Awareness Month. This research provides insights into the online conversations and trends related to oral cancer awareness. It highlights the effective utilization of social media platforms like Twitter for promoting awareness and disseminating information about oral cancer.

## Materials and methods

We collected English public tweets from April 1, 2022, through April 30, 2022, to examine the pattern and origin of awareness activities during Oral Cancer Awareness Month using the Twitter public streaming Application Programming Interface (API). We used various keywords, hashtags, and phrases to search for Twitter mentions of oral cancer. These search terms included OralCancerAwarenessMonth, tonguecancer, headandneckcancer, oralcancer, mouthcancer, oral cancer screening, OralCancerScreenings, Oraltumor, oral care, survivorship oral, oral cancer screening, self-oral examination, self-mouth examination, oralcancertreatments, oralcancerawareness, oralcancersurvivor, oralcancersurvivors, oralcancerprevention, and oralcancerdetection. We retrieved and analyzed all tweets’ content, including URLs, images, and videos, and excluded all non-English tweets. Additionally, we also eliminated duplicate tweets, mentions, retweets, reply to tweets from analysis to ensure that the dataset only involved original contents. All tweets were saved in a CSV file.

After the identification of tweets related to oral cancer, conversation data were analyzed and stratified quantitively using hashtags and words. To prepare the text data for analysis, several preprocessing steps were undertaken using Natural Language Toolkit (NLTK) techniques. These steps included lemmatization, stemming, stop word removal, and regular expression filtering to eliminate symbols and unnecessary keywords. Next, we employed the BERTopic word model technique. This approach leveraged transformers and c-TF-IDF (contextualized Term Frequency-Inverse Document Frequency) to generate easily interpretable topics. Additionally, word clouds were created to visually represent the top words based on their c-TF-IDF scores. In these word clouds, the size of each word corresponds to its score, with larger sizes indicating higher scores to highlight the most significant and relevant words associated with oral cancer awareness. The origin of tweets was identified and the distribution of tweets among continents was plotted on the map using datawrapper software.

We classified users as individual or organization using a new text-mining approach applied to English-only tweets. We further classified and verified users based on their Twitter profile descriptions, excluding duplicate and unverifiable profiles. Around 628 users were not verified and classified.

The statistical analysis was descriptive using Microsoft Excel software to calculate the frequency and percentage of various factors within the collected data. The analysis was conducted to know about tweet distribution among countries and the prevalence of participants on Twitter during Oral Cancer Awareness Month.

## Results

We extracted a total of 5557 tweets according to keywords and hashtags for oral cancer, and 5543 tweets met the inclusion criteria. Of these, 3850 (69.5%) tweets were by individuals, 1,065 (19.2%) by organizations, and 628 (11.3%) by unidentified users. Out of the total number of unique individual tweets, 23.9% (922 tweets) indicated that they work in non-health-care fields. The health-care providers and physicians were 19.6%, and only 31 (0.8%) tweets were identified as being from dentists. Of 1065 tweets from organizations, 9.7% were from hospitals and health-care-related parties, 7.3% from cancer-related foundations, and 7.1% from oral health-care-related organizations (Table 1).

**Table 1:** Distributions of tweets among individuals and organizations The data has been represented as frequency (N) and percentage (%).

Individual tweets	N (%)	Organization tweets	N (%)
Miscellaneous person	1550 (40.3)	Miscellaneous	680 (63.8)
Scientist, researcher, educator	388 (10.1)	Oral health care (dental clinics, services, society, group)	76 (7.1)
Non-health-related individuals	922 (23.9)	Research-related center, group, or company	74 (6.9)
Physicians and other health-care providers	756 (19.6)	Cancer-related foundation, center, group, society	78 (7.3)
Student (undergraduate, PhD )	105 (2.7)	Journal or publisher	54 (5.1)
Patients, survivor, fighter	129 (3.4)	Hospitals, health-care-related services, association, society	103 (9.7)

**Figure 1:**
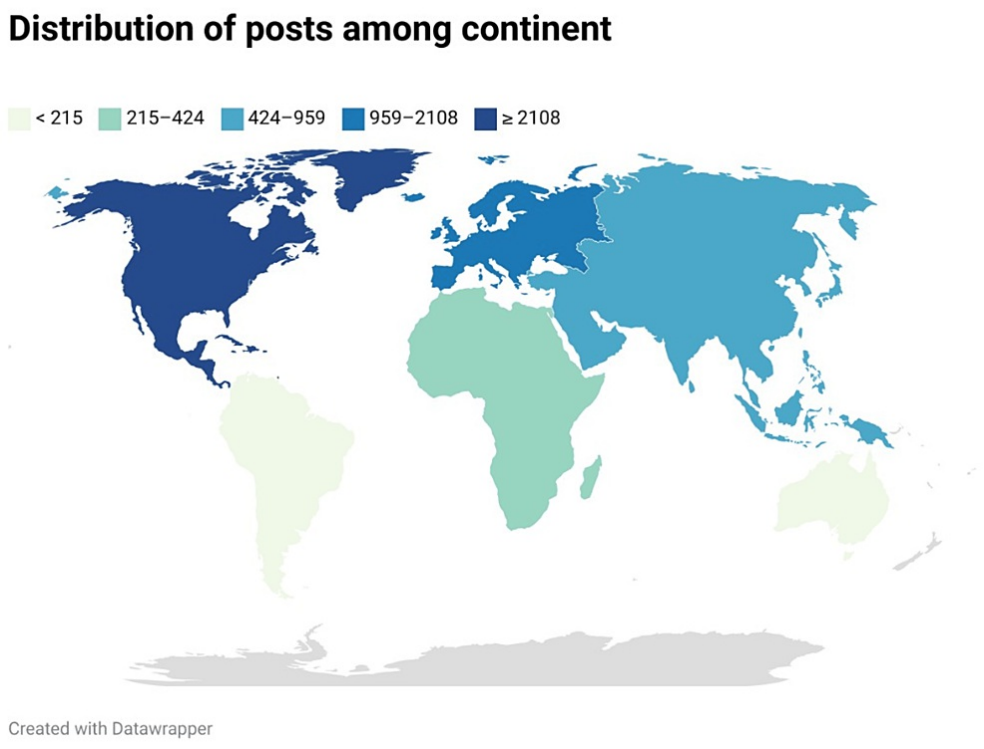
Distribution of tweets among continents for Oral Cancer Awareness Month (April 2022) The data has been represented as frequency (N) and the map was created by the author using datawrapper.

**Figure 2:**
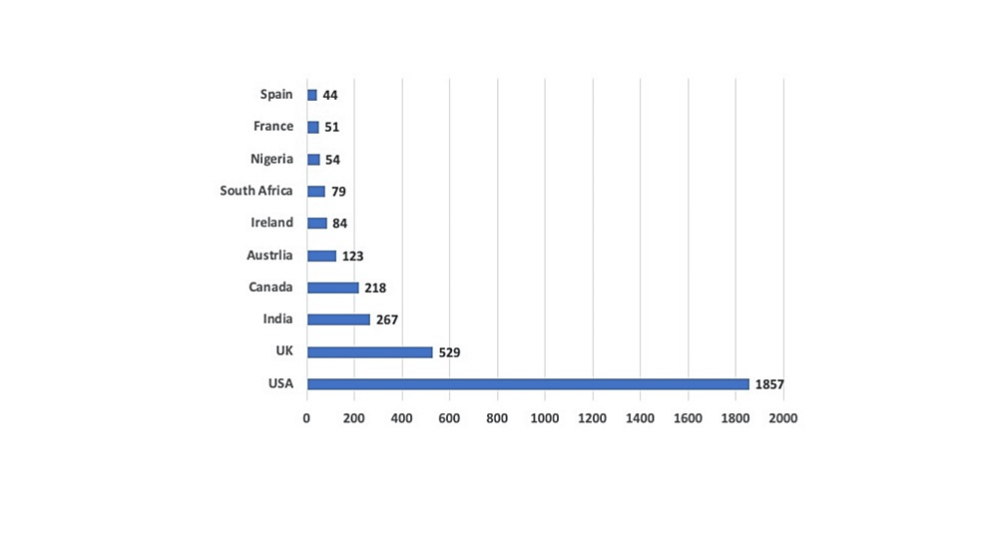
Top 10 countries with tweets during Oral Cancer Awareness Month The data has been represented as frequency (N).

Most of the tweets were from North America (2108), and the United States (US) was the top participant with 47.9% of country-tracked tweets. The lowest participating continents were Australia and South America with 129 and 42 tweets, respectively (Figure 1). The most common countries posted during Oral Cancer Awareness Month are illustrated in Figure 2.

**Figure 3:**
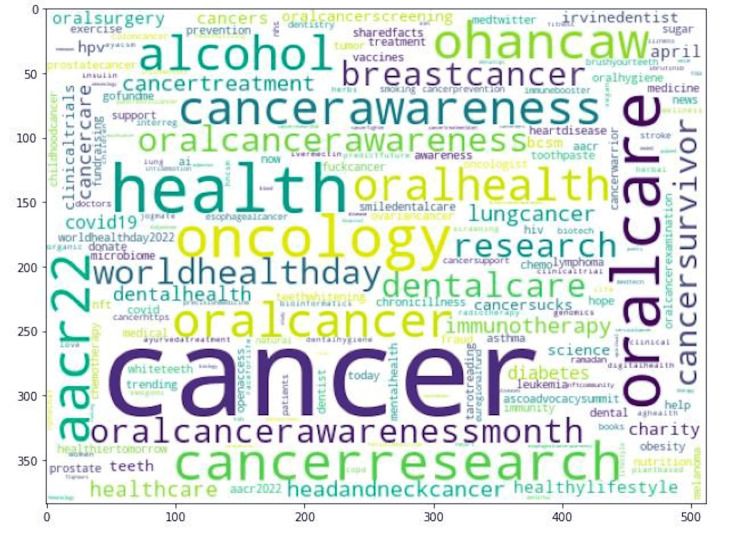
Top word counts in tweets during Oral Cancer Awareness Month The size of each word in the word cloud is determined by its frequency. The words that appeared more frequently will be displayed larger in font size.

Word cloud visualization is an efficient method for analyzing sentiment distribution. We evaluated the frequencies of all words in a given text corpus. A higher frequency count means that words appear more frequently in the corpus. The resulting word cloud displays this information by showcasing the size of each word based on its frequency in the corpus. As a result, words that are used more frequently will stand out in the word cloud with larger and bolder font (Figure 3). The tweets covered a variety of topics, from cancer and oral health to oncology, cancer research, cancer awareness, and even alcohol.

## Discussion

The objective of our study was to analyze the use of Twitter during Oral Cancer Awareness Month, a yearly event observed globally in April. This awareness month aims to raise awareness about oral cancer, including its risk factors, prevention, early detection, and treatment. We extracted 5543 tweets about oral cancer, and our analysis revealed various topics such as cancer, oral care, oncology, cancer research, cancer awareness, and alcohol. A similar study for laryngeal cancer found that HPV, diagnosis, early detection, and treatment options were the most common theme. In our study, we observed that the oral cancer network on Twitter appeared to be more popular than other types of cancer, considering the volume of tweets in a specific period of time [20].

The vast majority of oral cancer-related top posts were written by anonymous users who could not be categorized due to unclear profile information. Most tweets were driven by individuals who were not affiliated with health-care professionals (23.9%), followed by health-care providers, including physicians (19.6%), and then by researchers and scientists (10.1%). Dentists constituted only 0.4% of the participants on Twitter during the month. However, as the use of social media for health information continues to grow, increased awareness and the presence of dentists on these platforms can potentially benefit patients. Participation of non-health-care individuals and organizations in oral cancer awareness on Twitter has a significant impact on public awareness because it reaches a wide audience, amplifies messages through retweets and hashtags, and provides relatable experiences. Their involvement brings diverse perspectives and creative ideas, which enhance engagement and awareness about oral cancer. We found that organizations were less active in tweeting about oral cancer than individuals within a one-month time frame. This finding aligns with previous research that specifically focuses on breast cancer prevention and demonstrates higher engagement from individuals on Twitter during Breast Cancer Awareness Month compared to organizations [16]. However, it is important to note that both organizations and individuals have different impacts and can convey distinct messages in their tweets.

Social media platforms like Twitter are highly suitable for facilitating instant and individual communication between individuals from diverse backgrounds and experience levels. The use of Twitter varies worldwide, and different populations participate to different degrees. Unfortunately, many people cannot take advantage of Twitter because of geographic, financial, and/or political constraints. In our study, most tweets originated from North America, especially the US (33.5%), followed by Europe and the United Kingdom (9.5% of all tweets). Another study for cervical cancer showed that most tweets also originated from the United Kingdom (62.4%) and the US (28.7%), while India’s participation was 4.8%. Acknowledging that English is the primary language in these countries helps us understand the potential factors that may have contributed to the higher volume of tweets from these regions. Other countries may not increase their awareness activity during Oral Cancer Awareness Month (April) or Twitter is possibly not a common social media platform in some countries. To enhance the global reach of an oral cancer awareness campaign, it is important to encourage countries from different regions, especially South America, Australia, Africa, and Asia to actively engage in promoting oral cancer awareness and education.

However, there are certain limitations to consider in this study. Within the dataset, some tweets contained a mixture of English and non-English characters. While our study focused on collecting English tweets, this reliance on a specific language introduces a potential bias. By excluding non-English tweets, we may not capture the full range of discussions and perspectives on oral cancer awareness, limiting the generalizability of our findings to English-speaking populations. Additionally, given the lack of user-provided descriptions in their profiles, we encountered challenges in classifying all users. Another limitation of the study is the possibility of bias in the data collection, despite using a comprehensive set of relevant keywords and hashtags. Finally, it is widely recognized that Twitter users are not a fully representative sample of the general population. Therefore, Twitter data should be interpreted with caution and should not be generalized.

We believe that Twitter will remain a highly active platform for cancer awareness communication. Future studies in oral cancer awareness on social media can explore various directions, such as comparing different platforms and languages to understand their unique characteristics and effectiveness, conducting longitudinal evaluations to monitor the progress of awareness campaigns over time, and assessing the impact of shared content on social media.

## Conclusions

Twitter can be a valuable tool to expand the reach of the oral cancer message and maximize the potential of marketing. The use of a social media platform could demonstrate that the public can be ill-informed along with providing correct information, depending on how social media regulates that information. Furthermore, health organizations must enact laws and rules to regulate social media information that promotes and advertises dangerous products or misleading information. Through the strategic use of social media communication, including education and training in this area, researchers and health-care professionals can contribute to improving the social media environment for crucial topics like cancer.
